# Adiponectin Mediates the Protection of H_2_S Against Chronic Restraint Stress-Induced Cognitive Impairment *via* Attenuating Hippocampal Damage

**DOI:** 10.3389/fnbeh.2021.623644

**Published:** 2021-05-05

**Authors:** Qiong-Yan Tang, Min Li, Lei Chen, Jia-Mei Jiang, Sheng-Lan Gao, Fan Xiao, Wei Zou, Ping Zhang, Yong-Jun Chen

**Affiliations:** ^1^Department of Neurology, The Affiliated Nanhua Hospital, University of South China, Hengyang, China; ^2^Hengyang Key Laboratory of Neurodegeneration and Cognitive Impairment, Institute of Neuroscience, Hengyang Medical College, University of South China, Hengyang, China; ^3^Institute of Neurology, The First Affiliated Hospital, University of South China, Hengyang, China

**Keywords:** adiponectin, hydrogen sulfide, chronic restraint stress, cognitive impairment, hippocampal damage

## Abstract

Emerging evidence shows that chronic restraint stress (CRS) can induce cognitive dysfunction, which involves in hippocampal damage. Our recent research reveals that hydrogen sulfide (H_2_S), a novel gasotransmitter, protects against CRS-induced cognitive impairment, but the underlying mechanism remains unclear. Adiponectin, the most abundant plasma adipokine, has been shown to elicit neuroprotective property and attenuate cognitive impairment. Hence, the present work was aimed to explore whether adiponectin mediates the protective effect of H_2_S on CRS-induced cognitive impairment by inhibiting hippocampal damage. Results found that administration of Anti-Acrp30, a neutralizing antibody of adiponectin, obviously reverses sodium hydrosulfide (NaHS, an exogenous H_2_S donor)-induced the inhibition on CRS-induced cognitive impairment according to Y-maze test, Novel object recognition (NOR) test, and Morris water maze (MWM) test. In addition, Anti-Acrp30 blocked the protective effect of NaHS on hippocampal apoptosis in rats-subjected with CRS as evidenced by the pathological changes in hippocampus tissues in hematoxylin and eosin (HE) staining and the increases in the amount of the condensed and stained to yellowish-brown or brownish yellow neuron nucleuses in terminal deoxynucleotidyl transferase transfer-mediated dUTP nick end-labeling (TUNEL) staining as well as the expression of hippocampal pro-apoptotic protein (Bax), and a decrease in the expression of hippocampal anti-apoptotic protein (Bcl-2). Furthermore, Anti-Acrp30 mitigated the inhibitory effect of NaHS on CRS-induced oxidative stress as illustrated by the up-regulation of malondialdehyde (MDA) content and the down-regulation of superoxide dismutase (SOD) activity and glutathione (GSH) level in the hippocampus. Moreover, Anti-Acrp30 eliminated NaHS-induced the reduction of endoplasmic reticulum (ER) stress-related proteins including binding immunoglobulin protein (BIP), C/EBP homologous protein (CHOP), and Cleaved Caspase-12 expressions in the hippocampus of rats-exposed to CRS. Taken together, these results indicated that adiponectin mediates the protection of H_2_S against CRS-induced cognitive impairment through ameliorating hippocampal damage.

## Introduction

Pertaining to stress, it is an obvious prevalent experience that a large number of individuals are facing different levels of chronic stress in life (Cohen et al., 2016; Yaribeygi et al., 2017). In fact, there is growing evidence reveals that chronic stress is a major risk factor for several human disorders, such as Alzheimer’s disease and cognitive dysfunction, which is closely associated with the impairments of hippocapal neurogenesis, learning and memory, and emotional responses (McEwen, 2017; Lupien et al., 2018). Chronic restraint stress (CRS), as a typical model to simulate a living state of unpredictable setbacks in our daily life, also has ability to exacerbate neurodegeneration and impair cognitive function through inhibiting hippocampal neural activity, attenuating synaptic plasticity, and reducing neuronal cell survival (Anibal et al., 2017; Ngoupaye et al., 2017; Samarghandian et al., 2017). Available evidence shows that CRS causes the damage of hippocampal-dependent spatial learning and memory related to stress-caused synaptic dysfunction and oxidative damage, endoplasmic reticulum (ER) stress cascade and apoptosis in the hippocampal neurons (P. Said Mohammadi et al., 2014; Huang et al., 2015; Jangra et al., 2017). Therefore, it is urgent and essential to find the therapeutic regimens of hippocampal damage and cognitive impairment induced by CRS, which is beneficial to improve human health.

Hydrogen sulfide (H_2_S) is a novel integral endogenous signaling gasotransmitter molecule with a broad range of physiological and pathophysiological functions, such as inhibiting apoptosis and oxidative stress, reducing ER stress, improving synaptic plasticity, and promoting hippocampal memory formation in the neurodegenerative diseases (Kamat et al., 2015; Nagpure and Bian, 2015; Sandesh et al., 2016). Mounting evidence has revealed that H_2_S exerts protection against cognitive dysfunction (He et al., 2019; Li et al., 2019). In addition, our team also demonstrates that H_2_S ameliorates CRS-induced cognitive impairment and hippocampal injury (Xiao-Na et al., 2017). However, the underlying mechanisms by which H_2_S attenuates CRS-induced cognitive impairment have not been fully elucidated.

Adiponectin, an endogenous bioactive peptide protein, is secreted by adipocytes (Fang and Judd, 2018; Z. V. Wang and Scherer, 2016). Studies have revealed the physiological roles of adiponectin in inflammation, apoptosis, oxidative stress, obesity, diabetes, and atherosclerosis *via* interaction with its two receptors (AdipoR1 and AdipoR2) (Achari and Jain, 2017; Andel et al., 2018; Forny-Germano et al., 2019). Besides to adipocytes, evidence has also elucidated that adiponectin is also detected in cerebrospinal fluid (Katarina et al., 2007) and adiponectin receptors are expressed widely in the central nervous system (Thundyil et al., 2012). Adiponectin influences brain functions, such as energy homeostasis, hippocampal neurogenesis, and synaptic plasticity, eliciting neuroprotective (Thundyil et al., 2012; Jenna et al., 2018). Notably, growing research confirms that adiponectin participates in regulating the development of cognitive impairment (Fujita et al., 2018; Forny-Germano et al., 2019; T. F. Huang et al., 2019). It has been shown that the decreased level of adiponectin is associated with the degree of cognitive impairment (T. F. Huang et al., 2019) and increasing adiponectin level can restore hippocampal neurogenesis impairment and improve cognitive functions in Alzheimer’s disease mice (Ng et al., 2016). These findings implied that adiponectin plays critical roles in improving cognitive function. In addition, our previous studies demonstrate that H_2_S up-regulates the expression of adiponectin in the hippocampus of CRS-exposed rats (Tian et al., 2018). Therefore, the present study further investigated whether adiponectin-attenuated hippocampal injury is inseparable from the protection of H_2_S against CRS-induced cognitive impairment.

In the present work, we demonstrated that neutralizing adiponectin by Anti-Acrp30 not only blocks the protective effect of NaHS (an exogenous H_2_S donor) on CRS-induced cognitive impairment, but also reverses NaHS-induced the inhibition on hippocampal apoptosis, oxidative stress and ER stress in CRS-treated rats. These results indicated that adiponectin mediates H_2_S-antagonized CRS-induced cognitive impairment *via* inhibiting hippocampal damage.

## Materials and Methods

### Reagents

Sodium hydrosulfide (NaHS, a donor of H_2_S) was purchased from Sigma (St. Louis, MO, United States). Anti-Acrp30 was supplied by Santa Cruze Biotechology (CA, United States). The malondialdehyde (MDA) enzyme-linked immunosorbent assay (ELISA) kit was supplied by Uscn Life Science, Inc. (Wuhan, China). The glutathione (GSH) enzyme-linked immunosorbent assay (ELISA) kit was obtained from Bio-Swamp Life Science, Inc. (Wuhan, China). Nitro-Blue-Tetrazolium (NBT) kit was obtained from Beyotime Institute of Biotechnology (Shanghai, China). The Bicinchoninic Acid (BCA) Protein Assay Kit was obtained by Dojindo Molecular Technologies, Inc. (Rockvile, MD, United States). The primary antibodies against Bip, Chop, Cleaved Caspase-12, Bcl-2 and Bax were purchased from Cell Signaling Technology (Boston, MA, United States). The deoxynucleotidyl transferase transfer-mediated dUTP nick end-labeling (TUNEL) staining kit and hematoxylin and eosin (HE) staining kit were purchased from KeyGEN BioTECH (Nanjing, China).

### Experiment Animals

Fifty adult male Sprague–Dawley (SD, 200–220 g, 6 weeks) rats were purchased from Hunan SJA Laboratory Animal Co., Ltd (Changsha, China), and housed under standard laboratory conditions (a normal 12 h light/dark cycle, a room temperature of 22 ± 1°C with relative humidity of 55% ± 5%) with free access to food and water. All the experiment were censored and authorized by the State Science and Technology Commission of China and were approved by the Ethical Committee of University of South China.

### Chronic Restraint Stress Procedure

Chronic restraint stress can injure hippocampal-dependent spatial learning and memory (Takuma et al., 2007). The experiment procedure was performed and modified according to previous study (Q. Zhang et al., 2017). In brief, the stressed rats were restrained in 50 ml stainless steel tubes to limit their autonomous actions for 6 h/day (from 9: 00 p.m. to 15: 00 a.m.) for consecutive 28 days. The control rats remained in their home cages without stress exposure.

### Experimental Design and Drug Administration

**FIGURE 1:**
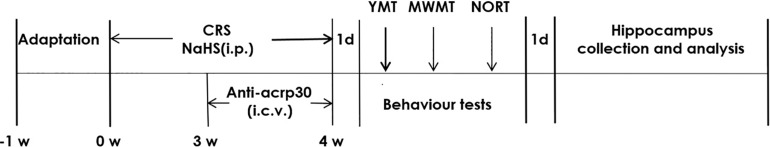
Schematic diagram of the experimental procedures. CRS, chronic restraint stress; YMT, Y-maze test; MWMT, Morris water maze test; NORT, novel object recognition test; i.p., intraperitoneal injection; i.c.v., intracerebroventricular injection.

After 7 days of acclimation, all rats (10 rats in each group) were randomly divided into five groups: Control group, rats were treated without stress and injected with equal amount of PBS (I.P.) as NaHS group for 4 weeks and intracerebroventricularly injected (i.c.v.) with equal amount of artificial cerebrospinal fluid (ACSF) as Anti-Acrp30 group in the last 7 days; CRS group, rats were fixed 6 h/day from 9.a.m to 15 p.m for 28 days in 50 ml stainless steel tubes and simultaneously given PBS (I. P.) for 4 weeks; NaHS (100 μmol/kg) + CRS group, rats were exposed to CRS and co-injected with NaHS (100 μmol/kg, i.p.) for 4 weeks; NaHS (100 μmol/kg) + CRS + Anti-Acrp30 group: rats were exposed to CRS and co-injected with NaHS (100 μmol/kg, i.p.) for 4 weeks and Anti-Acrp30 (1 μg, i.c.v.) for the last week of the 4-week CRS procedure; Anti-Acrp30 (1 μg) alone group: rats were injected with Anti-Acrp30 (1 μg/day, 7 days, i.c.v.). All behavior tests were performed after 24 h of the last injection (Supplementary Material). On the next day of all behavior tests, all rats were killed and the hippocampus region tissues were rapidly removed and stored at −80°C for analysis (Figure 1). There were a total of 50 rats at the start of the experiment. And at the end of behavior tests, there were 10 rats left in the control group, eight rats left in the CRS group, NaHS + CRS group and NaHS + CRS + Anti-Acrp30 group, as well as nine rats in the Anti-Acrp30 alone group, we analyzed that improper injection of drugs and accidental death in behavioral experiments were the causes of death in rats.

### Intracerebroventricular Injection (Lateral Ventricular Injection)

All rats were deeply anesthetized with 1% sodium pentobarbital (0.04 ml/kg, i.p.) and fastened to a stereotaxic apparatus for operation below the center of the ear pole. Pre-exposure of the bregma completely, the positioning coordinates of the needle insertion point are as follows: AP: 1.0 mm, R or L: 1.5 mm. The lateral ventricle of SD rats was fed with a single cage for 7 days to prevent the entrapment injury and the transmission of infection. Three days after operation, rats were given penicillin 200.000 units intraperitoneal daily to prevent infection. Anti-Acrp30 (1 μg/day) was injected into the lateral ventricle with an injection rate of 0.75 μL/min and left the needle for 2 min.

### Y-Maze Test

Y-maze is major test used to assess short-term spatial recognition memory and locomotor activity (Willi et al., 2012). The apparatus consists of three arms with a size of 90 cm × 90 cm × 70 cm joined in a central area in the form of an equilateral 120° angle. Three arms are randomly set up: A arm, B arm, and C arm. After each experiment, the Y-maze was scrubbed with high concentration alcohol to prevent the residual odor from disturbing the experiment. Rats were placed in the central area of the Y-maze and then allowed to move freely for 5 min, and the experimenter would record the sequences as correct without repeated entry into each wall at a time (such as ABC, ACB, and BCA etc.). The spontaneous alternation rate = [(total correct alternating sequence/total alternating sequence) × 100%] of rats was calculated as an index of spatial cognitive ability. At the same time, the total number of rats entering each arm was recorded as an index of movement ability.

### Novel Object Recognition Test

Novel object recognition (NOR) test is used to evaluate the hippocampal-related cognitive modification in rodent (Cohen and Stackman, 2015). The NOR test is consisted of three parts: the habituation phase, training phases and test period. The white plastic box (50 cm × 40 cm × 30 cm) was used in the new object recognition experiment. In the habituation phase, 2 days before training, rats were placed into the empty behavioral box (50 cm × 50 cm × 60 cm) without any items and allowed to move for 5 min in order to adapt to the surrounding environment. The exploratory behavior was taken into consideration when rats smell and touch the objects. In the training period, two cylindrical objects (a) identical in shape, size, and color were placed in opposite corners of the box, and the rats were placed in the middle of the box and allowed to move freely for 5 min to explore these two objects. In the test period, a novel cubic object (b) with different size and color was used to replace one of the two identical cubes, and rats were also allowed to explore two objects for 5 min freely. The whole experiment process was recorded by video. After each experiment, 70% ethanol was used to clean objects to ensure that odor cues did not a behavior. The interval of the training period and testing period is 2 h. The time of exploring the cylindrical objects (a) and the cubic objects (b) was recorded respectively. The discrimination index [(DI, DI = (b-a)/(a + b)] and the recognition index [(RI), RI = b/(a + b)] were calculated to determine the cognitive function of rats.

### Morris Water Maze Test

The Morris water maze (MWM) test is applied to analysis the spatial learning memory and working memory of rats (D’Hooge, 2001). It is composed of water maze device, software analysis system and image collection of water maze. The water maze device consists of a circular container (120 cm in diameter, high 50 cm) filled with water (25 ± 2°C, 35 cm deep) where non-fatty dry milk was added to make the water opaque, and divided into four quadrants. A simple movable escape platform (diameter 10 cm) was placed in the center of a target quadrant 2 cm below the surface of the water. The water maze experiment was divided into three processes: the acquisition period, the probe period, and the visual platform period. In the acquisition period: rats were put into the position from target quadrant and allowed freely to locate the hidden platform in the target quadrant within 120 s. this process was conducted four times per day, and 20 min between two times for 5 days. The time of finding the underwater escape platform within 120 s was recorded (defined as incubation period), while if rat failed to find the platform within 120 s, it was guided to the platform and allowed to stay there for 20 s. After every experiment, dried the rats, put them back into the cage, and cleaned up the sundries in the pool. In the probe trial period: 1 day after the repeated acquisition trial, the platform was removed and. The contralateral quadrant of the previous escaped quadrant was used as the starting quadrant, each rat was place headed toward the wall for 120 s of exploratory training. The swimming time spent in the target platform quadrant, the number of times entering the target platform area and the number of times swimming in the ring were recorded, which were used as an index to detect the learning memory and working memory of the rats. In the visual platform experiment: in order to eliminate the influence of visual and motor ability on the above results. After the probe trial, the escape platform was placed in the contralateral quadrant of the acquisition trial, which was higher than the horizontal level of 2 cm. The rats were placed in water from the opposite quadrant of the platform to record the path and time of reaching the platform.

### Determination of MDA Content, SOD Activity, and GSH Level

Hippocampal homogenate was obtained and centrifuged at 12,000 *g* for 10 min. The supernatant was collected and the total protein content was detected by BCA protein assay. Hippocampal lipid peroxidation was estimated by detecting MDA content using a MDA ELISA kit on the basis of previously described protocol (Tsikas, 2017). The activity of SOD in hippocampus was determined by NBT kit according to the procedure previously described (Rodríguez-Martínez et al., 2013). The level of GSH in hippocampus was tested by a GSH ELISA kit. The detailed steps were all based on the manufacturer’s instructions for the kits that has been described elsewhere (Akang, 2019). The results were expressed as μg/mg protein.

### Western Blot Analysis

Western blot analysis is based on previous classical experimental process (Death and Elisa, 2014). Hippocampus was homogenized with ice-cold homogenate buffer containing 1% (v/v) protease inhibitors and 1% (v/v) phosphatase inhibitor (Sigma-Aldrich Co., MO, United States) and incubated on ice for 30 min. After centrifugation at 12,000 × *g* for 10 min at 4°C, the supernatant of the hippocampal homogenate was collected and the concentration of total protein was detected by BCA Protein Assay Kits. The equal amount of protein (30 μg) was separated on 12% SDS-PAGE and electrotransferred to polyvinylidene difluoride (PVDF) membrane. After blocking with 5% non-fat milk for 2 h at room temperature, the membranes were incubated with the primary antibodies against Bip (1:1,000), Chop (1:1,000), Cleaved Caspase-12 (1:1,000), Bcl-2 (1:2,000), Bax (1:2,000), and β-actin (1:2,000) at 4°C overnight. β-actin was used as an internal control. Then, the membranes were washed three times with TBST (Tris-Burffered Saline and Tween20, 50 mmol/L Tris–HCl, pH 7.5,150 mmol/L NaCl, 0.1% Tween-20) for 10 min and incubated with 1:5,000 diluted anti-rabbit second antibody (1:5,000) for 2 h at room temperature. Finally, the band was visualized by enhanced chemiluminescence system (BeyoECL Plus kit, Beyotime, P0018). Protein level was quantified using Image J software (National Institutes of Health, MD, United States) and was normalized to β-actin.

### Hematoxylin and Eosin Staining

The morphological changes of hippocampal tissues were observed by HE staining under standard procedure (Rodríguez-Martínez et al., 2013). On the next day of all behavior tests, a total of four rats were transcardially perfused with 4% paraformaldehyde in each group. Hippocampal tissues were softly removed, and then immerged in the fixative solution for 24 h. The hippocalpal tissue was cut into 5 μm slices, then the slice were added to xylene I for 10 min, xylene II for 10 min, ethyl alcohol I for 5 min, ethyl alcohol II for 5 min, 95% alcohol for 5 min, 90% alcohol for 5 min, 70% alcohol for 5 min and 70% alcohol for 5 min and washed in running water. After that, the slice were stained with hematoxylin for 4 min and eosin for 3 min. in addition, added to 95% alcohol I, 95% alcohol II for 5 min, ethyl alcohol I for 5 min, ethyl alcohol II for 5 min, xylene I for 10 min, and xylene II for 10 min and sealed with neutral gum. The pathological changes were observed by light microscope.

### Terminal Deoxynucleotidyl Transferase Transfer-Mediated dUTP Nick End-Labeling Staining

The apoptosis of hippocampal cells was observed by TUNEL staining. One day after the end of the behavioral tests, the head of rats were anesthetized by intraperitoneal injection of sodium pentobarbital (45 mg/kg), the head was cut down, removed and washed the hippocampus of rats. Hippocampal tissue sections of each rat were embedded in paraffin and dewaxed to water, incubated in H_2_O_2_ for 40 min to eliminate endogenous peroxidase activity, then incubated with protease K for 10 min, incubated with TUNEL reaction solution for 1 h, POD-transforming solution for 30 min, and DAB for 10 min. Then, hematoxylin was added for redyeing, dehydration, transparent and sealing. The neuron-positive markers of TUNEL staining were the condensed and stained to yellowish-brown or brownish yellow neuron nucleuses under microscope (× 400). The neuronal apoptosis index = (TUNEL positive neurons/total neurons) × 100%.

### Statistical Analysis

All the data were analyzed by SPSS software version 18.0 (SPSS Software, Inc., Chicago, IL, The United States). All data are presented as the mean ± SEM. The significance of intergroup differences was assessed by One-way ANOVA followed by the Bonferroni test for *post hoc* analysis. *P* < 0.05 was considered statistically significant.

## Results

### Anti-Acrp30, a Neutralizing Antibody of Adiponectin, Abolishes the Protective Effect of H_2_S on CRS-Induced Cognitive Impairment in Y-Maze Test

**FIGURE 2:**
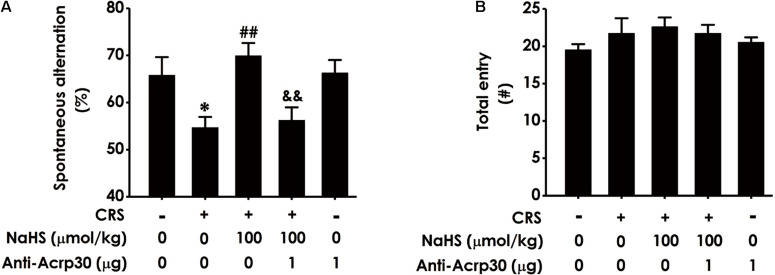
Effects of Anti-Acrp30 on NaHS-resulted in the improvement of CRS-induced cognitive impairment in Y-maze test. Rats were cotreated with NaHS (100 μmol/kg/day, i.p.) and CRS (6 h/day) for 3 weeks, and then co-administrated with Anti-Acrp30 (1 μg/day, i.c.v.) for further 1 week. The spatial working memory function of rat was detected by the Y-maze test and the alternation performance **(A)** and the total arm entries **(B)** were respectively recorded. All values are expressed as mean ± SEM (*n* = 8–10/group). *P < 0.05, vs. control group; ^##^*P* < 0.01, vs. CRS-exposed alone group; ^&&^*P* < 0.01, vs. cotreatment with NaHS and CRS group. CRS: chronic restrain stress.

To confirm whether adiponectin mediates H_2_S-attenuated cognitive dysfunction in CRS-exposed rats, the effect of Anti-Acrp30 (1 μg/day, i.c.v., for 1 week), a neutralizing antibody of adiponectin, on cognitive function was assessed using Y-maze test, which utilizes the rodent’s innate tendency to explore a novel environment (Deacon and Rawlins, 2006). Results revealed that administration of NaHS (a donor of H_2_S, 100 μmol/kg/day, i.p., for 4 weeks) significantly attenuated CRS-induced the reduction of spontaneous alternation behavior in the Y-maze test (Figure 2A). However, treatment with Anti-Acrp30 markedly blocked NaHS-induced an increase in spontaneous alternation of rats co-treated with CRS. Additionally, there was no significant difference in the total numbers of entries into the arms among the five groups (Figure 2B), suggesting that Anti-Acrp30 did not affect locomotor activity. These data indicated that neutralization of adiponectin blocks the protective effect of H_2_S on CRS-induced spatial memory and working dysfunction.

### Anti-Acrp30 Blocks the Inhibition of H_2_S on CRS-Induced Cognitive Dysfunction in the Novel Object Recognition Test

**FIGURE 3:**
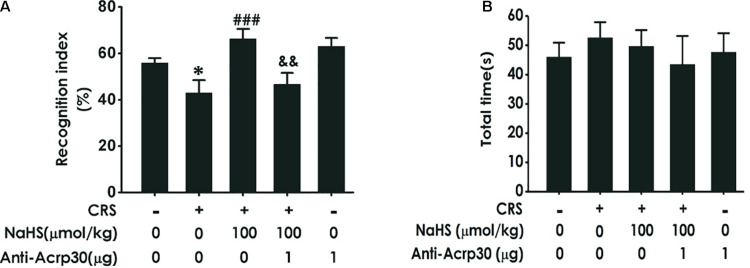
Effects of Anti-Acrp30 on NaHS-improved cognitive impairment in CRS-exposed rats in novel object recognition test. Rats were co-treated with NaHS (100 μmol/kg/day, i.p.) and CRS (6 h/day) for 3 weeks, and then co-administrated with Anti-Acrp30 (1 μg/day, i.c.v.) for further 1 week. The cognitive function was detected by the novel object recognition test and the discrimination index **(A)** and the total object exploration time **(B)** of rats were respectively recorded. All values are expressed as mean ± SEM (*n* = 8–10/group). *P < 0.05, vs. control group; ^###^*P* < 0.001, vs. CRS-exposed alone group; ^&&^*P* < 0.01, vs. co-treatment with NaHS and CRS group.

Then, the NOR test was used to determine changes in object recognition memory depending on the ability of animal to distinguish between familiar and novel objects. As illustrated in Figure 3A, treatment with NaHS (100 μmol/kg/day, i.p., for 4 weeks) significantly increased the time of rats spending exploring novel objects than familiar objects in CRS-exposed rats, while administration of Anti-Acrp30 (1 μg/day, i.c.v., for 1 week) obviously decreased the time of rats spending exploring novel objects than familiar objects in rats co-treated with NaHS and CRS. Importantly, there were no differences in the total exploration time among five groups (Figure 3B). These results also demonstrated that neutralizing adiponectin reverses the inhibitory effect of H_2_S on CRS-induced cognitive deficiency.

### Anti-Acrp30 Reverses the Ameliorative Effect of H_2_S on CRS-Elicited Cognitive Impairment in the Morris Water Maze Test

**FIGURE 4:**
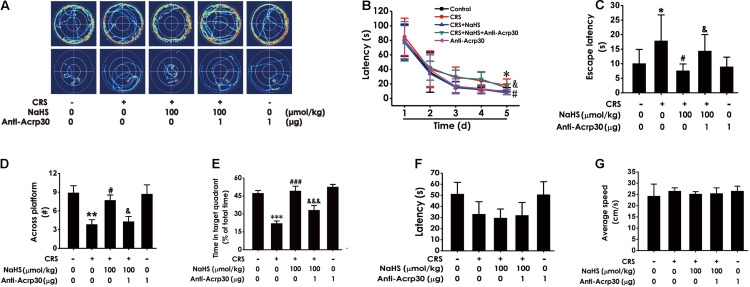
Effects of Anti-Acrp30 on NaHS-ameliorated cognitive dysfunction in CRS-exposed rats in Morris Water Maze test. Rats were co-treated with NaHS (100 μmol/kg/day, i.p.) and CRS (6 h/day) for 3 weeks, and then co-administrated with Anti-Acrp30 (1 μg/day, i.c.v.) for further 1 week. The route of rats in 1st and 5th in training phase **(A)**, the latency period during 5 days **(B)**, and the escape latency in 5th in the acquisition phase **(C)** were respectively recorded. The time of crossing the platform **(D)** and the proportionality of swimming time in target quadrant **(E)** in the probe trail were respectively recorded. The latency period **(F)** and the average speed **(G)** to reach platform in the visible platform test of rats were respectively recorded. All values are expressed as mean ± SEM (*n* = 6–7/group) in the captions of morris water maze test. *P < 0.05, **P < 0.01, ***P < 0.001 vs. control group; ^#^*P* < 0.05, ^###^*P* < 0.001 vs. CRS-exposed alone group; ^&^*P* < 0.05, ^&&&^*P* < 0.001 vs. co-treatment with NaHS and CRS group.

We further tested whether Anti-Acrp30 reverses the inhibitory effect of H_2_S on the CRS-exerted cognitive impairment in the MWM test. Results showed that NaHS (100 μmol/kg/day, i.p., for 4 weeks) treatment markedly decreased the escape latency in hidden-platform acquisition training (Figures 4A–C), increases the crossing platform times (Figure 4D), and promoted the percentage of time in the target quadrant in the probe trail (Figure 4E) in CRS-exposed rats. However, Anti-Acrp30 (1 μg/day, i.c.v., for 7 days) evidently reversed NaHS-induced the above amelioration in the rats. Importantly, all rats among five groups displayed similar latencies to the platform (Figure 4F) and average speed (Figure 4G) in the visible platform test, indicating that the rats in five groups have normal visual perception and swimming capability. Taken together, these data further suggested that neutralization of adiponectin eliminates the ameliorative effect of H_2_S on CRS-caused cognitive impairment.

### Anti-Acrp30 Abrogates the Protection of H_2_S Against CRS-Induced Apoptosis in the Hippocampus of Rats

**FIGURE 5:**
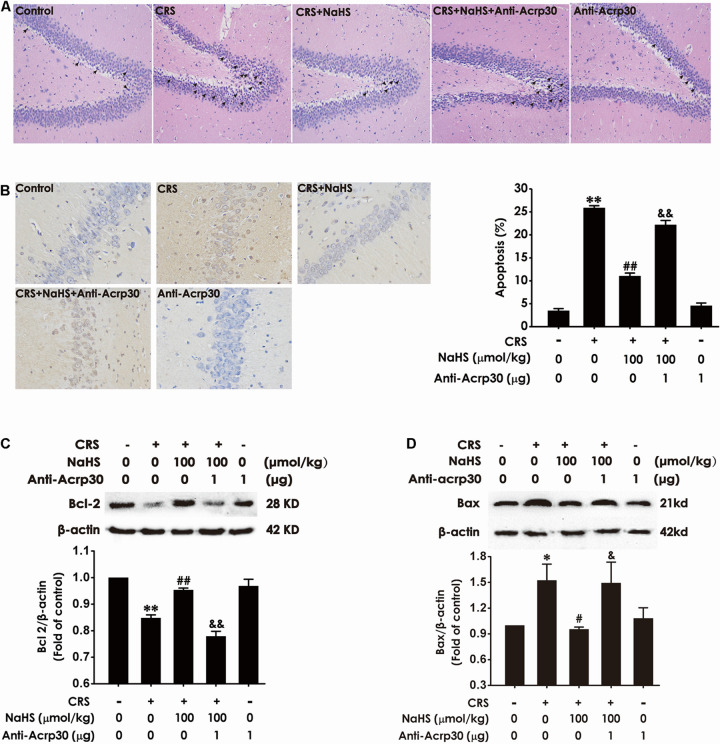
Effects of Anti-Acrp30 on NaHS-attenuated hippocampal apoptosis in CRS-exposed rats. Rats were co-treated with NaHS (100 μmol/kg/day, i.p.) and CRS (6 h/day) for 3 weeks, and then co-administrated with Anti-Acrp30 (1 μg/day, i.c.v.) for further 1 week. **(A)** The hippocampus lesion was analyzed by HE staining (magnification × 200). **(B)** The hippocampal neuronal apoptosis was analyzed by TUNEL staining (magnification × 400). The expressions of Bcl-2 **(C)** and Bax **(D)** in the hippocampus were measured by western blot analysis. Date are mean ± SEM (*n* = 3–4/group). *P < 0.05, **P < 0.01, vs. control group; ^#^*P* < 0.05,^ ##^*P* < 0.01, vs. CRS-exposed alone group; ^&^*P* < 0.05, ^&&^*P* < 0.01, vs. co-treatment with NaHS and CRS group.

Next, to further explore whether hippocampal injury is involved in the contribution of adiponectin to the protective effect of H_2_S on CRS-induced cognitive impairment, the effect of Anti-Acrp30 on apoptosis was detected. As shown in Figure 5A, CRS-treated rats showed pyramidal cells in hippocampus were karyopyknosis in HE staining, administration of NaHS (100 μmol/kg/day, i.p., 4 weeks) reversed this abnormal morphological change, whereas neutralizing the activity of adiponectin by Anti-Acrp30 reversed the protective effect of NaHS on the structural morphological disorder of pyramidal cells in CRS rat. Furthermore, administration of NaHS (100 μmol/kg/day, i.p., 4 weeks) significantly eliminated CRS-induced an increase in the amount of the condensed and stained to yellowish-brown or brownish yellow neuron nucleuses under microscope (×400), which was blocked by treatment with Anti-Acrp30 (1 μg/day, i.c.v., for 7 days) (Figure 5B). In addition, NaHS (100 μmol/kg/day, i.p., 4 weeks) also decreased the expression of hippocampal anti-apoptotic protein (Bcl-2) (Figure 5C) and increased the expression of hippocampal pro-apoptotic protein (Bax) (Figure 5D) in CRS-treated rats. However, these effects of NaHS were also reversed by Anti-Acrp30 (1 μg/day, i.c.v., for 7 days). Anti-Acrp30 alone had no effect on hippocampal apoptosis (Figure 5). These data demonstrated that neutralization of adiponectin abolishes the inhibition of H_2_S on CRS-resulted in hippocampal apoptosis.

### Anti-Acrp30 Reverses the Protection of H_2_S Against CRS-Induced Oxidative Stress in the Hippocampus of Rats

**FIGURE 6:**
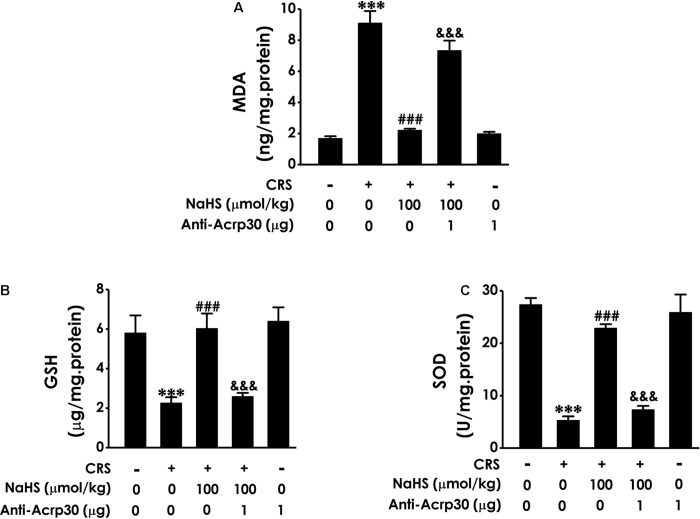
Effects of Anti-Acrp30 on NaHS-mitigated hippocampal oxidative stress in the CRS-treated rats. Rats were co-treated with NaHS (100 μmol/kg/day, i.p.) and CRS (6 h/day) for 3 weeks, and then co-administrated with Anti-Acrp30 (1 μg/day, i.c.v.) for further 1 week. **(A)** The content of hippocampal MDA was measured by malondialdehyde (MDA) enzyme-linked immunosorbent assay (ELISA) kit. **(B)** The level of hippocampal GSH was detected by glutathione (GSH) enzyme-linked immunosorbent assay (ELISA) kit. **(C)** The activity of hippocampal SOD was determined by NBT kit. Date are mean ± SEM (*n* = 3–4/group). ***P < 0.001, vs. control group; ^###^*P* < 0.001, vs. CRS-exposed alone group; ^&&&^*P* < 0.001, vs. co-treatment with NaHS and CRS group.

Our previous work have found that H_2_S can ameliorate CRS-induced hippocampal injury involved in attenuating oxidative stress (Xiao-Na et al., 2017). To further understand the underlying mediatory mechanisms of adiponectin in the protective role of H_2_S in CRS-induced cognitive dysfunction, the effect of Anti-Acrp30 on hippocampal oxidative damage was further investigated. Results revealed that treatment with NaHS (100 μmol/kg/day, i.p., 4 weeks) obviously decreased the level of MDA, a lipoxidation product (Figure 6A), increases the level of GSH (Figure 6B) and the activity of endogenous antioxidant enzyme SOD (Figure 6C) in the hippocampal of CRS-treated rats. However, these effects of NaHS were obviously attenuated by Anti-Acrp30 (1 μg/day, i.c.v., for 7 days). Anti-Acrp30 alone had no effect on oxidative stress in the hippocampus of rats compared with control group (Figure 6). These results indicated that neutralizing adiponectin abolishes the protection of H_2_S against CRS-elicited hippocampal oxidative stress.

### Anti-Acrp30 Blocks the Inhibitory Effect of H_2_S on CRS-Induced Hippocampal Endoplasmic Reticulum Stress in Rats

**FIGURE 7:**
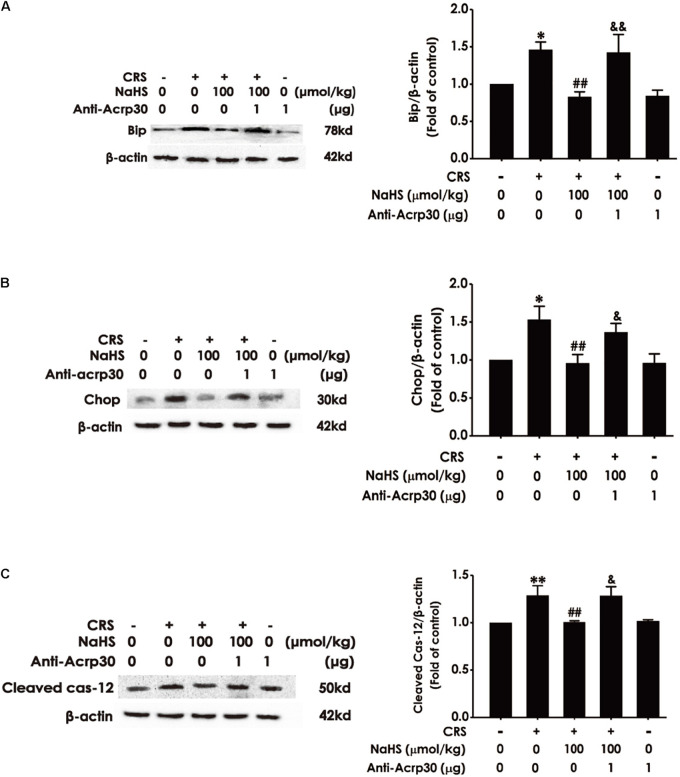
Effects of Anti-Acrp30 on NaHS-meliorated hippocampal ER stress in CRS-treated rats. Rats were co-treated with NaHS (100 μmol/kg/day, i.p.) and CRS (6 h/day) for 3 weeks, and then co-administrated with Anti-Acrp30 (1 μg/day, i.c.v.) for further 1 week. The expressions of hippocampal Bip **(A)**, Chop **(B)**, and Cleaved Caspase-12 **(C)** were measured by Western blot analysis. Date are the means ± SEM (*n* = 3–4/group). *P < 0.05, **P < 0.01 vs. control group; ^##^*P* < 0.01, vs. CRS-exposed alone group; ^&^*P* < 0.05, ^&&^*P* < 0.01, vs. co-treatment with NaHS and CRS group.

Next, to determine whether adiponectin is indispensable for the protection of H_2_S against CRS-induced ER stress, the impact of Anti-Acrp30 on the expressions of ER-associated proteins such as Bip, Chop, and Cleaved Caspase-12 were detected by western blot analysis. As shown in Figure 7, administration of NaHS (100 μmol/kg/day, i.p., for 4 weeks) obviously decreased the expressions of Bip (Figure 7A), Chop (Figure 7B) and Cleaved Caspase-12 (Figure 7C) in the hippocampus of CRS-exposed rats, while treatment with Anti-Acrp30 (1 μg/day, i.c.v., for 7 days) remarkably increased the expressions of Bip (Figure 7A), Chop (Figure 7B), and Cleaved Caspase-12 (Figure 7C) in the hippocampus of rats co-treated with NaHS and CRS, indicating that neutralizing adiponectin mitigates the inhibition of H_2_S on CRS-induced hippocampal ER stress.

## Discussion

The present work was to explore whether adiponectin mediates the protective effect of H_2_S on CRS-induced cognitive impairment *via* inhibiting hippocampal injury. Our results found that neutralizing adiponectin by Anti-Acrp30 reverses the protection of H_2_S against CRS-induced cognitive impairment. In addition, Anti-Acrp30 abolishes the inhibitory effect of H_2_S on hippocampal apoptosis, oxidative stress, and ER stress in the CRS-exposed rats, indicating that neutralizing adiponectin blocks H_2_S-induced the attenuation of hippocampal impairment in CRS-exposed rats. These data demonstrated that adiponectin mediates the antagonistic role of H_2_S in CRS-induced cognitive impairment by the suppression of hippocampal damage.

Research has confirms that H_2_S possesses essential functions in antagonizing cognitive impairment (J. Huang et al., 2020; Rizzo et al., 2020). Recently, our study has also proved that H_2_S also attenuates CRS-induced cognitive impairment (Xiao-Na et al., 2017), but the potential mechanism contributes to this process remains poorly understood. A growing number of studies have showed that adiponectin can promote cognitive function (J. Huang et al., 2020; Rizzo et al., 2020). Notably, we have previously found that NaHS (a donor of H_2_S) increases the level of adiponectin in the hippocampus of CRS-exposed rats (Ashish et al., 2018). These studies suggest that adiponectin may play a beneficial role in the anti-cognitive dysfunction of H_2_S. In the present study, we further found that neutralizing adiponectin by Anti-Acrp30 (Tian et al., 2018) attenuates the inhibitory effect of NaHS on CRS-induced cognitive impairment according to Y-maze test, NOR test, and MWM test. It should be explained that the inclusion of representative pathways of experimental groups in the probe trail of MWM test would make our results more comprehensive, which is what we need to pay more attention to in the following experiments. These results indicated that H_2_S-elicited inhibitory effect on CRS-induced cognitive impairment is mediated by adiponectin.

We further investigated the potential mechanisms that adiponectin mediates the antagonistic role of H_2_S in CRS-induced cognitive impairment. Previous studies have suggested that hippocampal apoptosis plays an essential role of cognitive impairment (Barun et al., 2011; Sun et al., 2020). CRS induces hippocampal lesions and learning and memory dysfunction associated with apoptosis (H. Zhang et al., 2020). Our previous studies reveal that exogenous H_2_S alleviates surgery- (Yin et al., 2020), formaldehyde- (Li et al., 2019), and diabetes- (Ma et al., 2017) induced cognitive dysfunction through inhibiting apoptosis in the hippocampus. Our recent study also reveals that H_2_S blocks CRS-led to cognitive impairment involved in attenuating hippocampal apoptosis (Xiao-Na et al., 2017). It has been demonstrated that adiponectin can alleviate hippocampal neuronal apoptosis (Lucassen et al., 2006; M. Wang et al., 2019). These studies indicate that adiponectin may contribute to the protective effect of H_2_S on CRS-induced cognitive impairment through inhibiting hippocampal apoptosis. In present study, our results further found that neutralizing adiponectin by Anti-Acrp30 abolishes the protection of NaHS against CRS-induced hippocampal apoptosis as evidenced by the increases in the amounts of pathological lesions in hippocampal neurons and the condensed and stained to yellowish-brown or brownish yellow neuron nucleuses under microscope (×400) and the expression of Bax as well as a decrease in the expression of Bcl-2. These results suggested that adiponectin mediates the beneficial effects of H_2_S against CRS-induced hippocampal apoptosis, thereby contributing to the protection of H_2_S against CRS-resulted in cognitive impairment.

Oxidative stress has been described in a variety of studies as one of the main pathways involved in the pathophysiology of cognitive impairment, such as CRS (H. Wang et al., 2020; H. Zhang et al., 2020). Our previous study demonstrates that H_2_S attenuates CRS-induced cognitive impairment by inhibition of hippocampal oxidative stress, but the protective mechanism of H_2_S on hippocampal oxidative stress have not been completely characterized (Xiao-Na et al., 2017). It has been reported that adiponectin plays an necessary role in preventing oxidative stress (Zhu et al., 2019). Adiponectin reduces oxygen-glucose deprivation-induced mitochondrial oxidative injury in hippocampal HT22 cells (Bodong et al., 2018). Adiponectin peptide also increased SOD and GSH-Px levels, and decreased ROS and MDA levels in glutamate-treated HT22 hippocampal neurons (Ye et al., 2018). Although there is no study on the antioxidant action of adiponectin under CRS and/or CRS-induced cognitive impairment, we hypothesized that adiponectin may mediate the protection of H_2_S against CRS-induced hippocampal oxidative stress according above researches. In the present, we first discovered that neutralizing adiponectin abolishes the protection of H_2_S against CRS-induced hippocampal oxidative stress as illustrated by the increases in MDA content, SOD activities, and GSH level in rats. These results indicated that adiponectin contributes to the protection of H_2_S against CRS-induced cognitive dysfunction *via* inhibiting hippocampal oxidative stress.

Endoplasmic reticulum stress is triggered by many physiological events and pathophysiological conditions, resulting from the accumulation of unfolded or misfolded intermediates in the ER (Zhu et al., 2019). Emerging evidences have proposed the contribution of ER stress to hippocampal injury and cognitive impairment (Ye et al., 2018). It has been reported that the levels of ER stress-related proteins including Bip, Cleaved Caspase-12 and Chop are increased in the brain of CRS-exposed rats (Jangra et al., 2016). In addition, our previous study reported that H_2_S attenuates CRS-induced the increases in the expressions of ER stress-related markers in the hippocampus of rats (Xiao-Na et al., 2017). It has also been reported that adiponectin plays an essential role in blocking ER stress (Hampe et al., 2017). To determine whether the adiponectin was necessary for H_2_S-inhibited hippocampal ER stress in CRS-exposed rats, we further evaluated the influence of Anti-Acrp30 on the expressions of ER stress related proteins in the hippocampus of rats cotreated with NaHS and CRS. The results found that Anti-Acrp30 obviously increased the expressions of Bip, CHOP and Cleaved Caspase-12 in the hippocampus of rats cotreated with NaHS and CRS, which indicated that inhibition of adiponectin abolishes the protection of H_2_S against hippocampal ER stress in CRS rats. This finding is consistent with the previous observations that adiponectin plays an important role in counteracting ER stress (Lutz et al., 2017). Based on the above results, our study suggested that adiponectin mediates the beneficial effect of H_2_S on CRS-induced hippocampal ER stress.

In summary, our present work demonstrated that neutralizing adiponectin by Anti-Acrp30 reverses the protective effect of H_2_S on CRS-induced cognitive impairment *via* inhibiting hippocampal apoptosis, oxidative stress, and ER stress. Combining our previous finding that H_2_S up-regulates the levels of adiponectin in the hippocampus of CRS-exposed rats (Tian et al., 2018), these results suggested that adiponectin mediates the anti-cognitive impairment effect of H_2_S in CRS-exposed rats through inhibition of hippocampal damage. The present finding provides a mechanistic explanation for the potential therapeutic value of H_2_S in the treatment of cognitive dysfunction.

## Data Availability Statement

The original contributions presented in the study are included in the article/Supplementary Material, further inquiries can be directed to the corresponding author/s.

## Ethics Statement

The animal study was reviewed and approved by the Ethical Committee of the University of South China.

## Author Contributions

J-MJ and Y-JC contributed to conception and design of the study. Y-JC, WZ, and PZ guided and supervised the study. Q-YT, ML, and LC performed the experiments. S-LG and FX analyzed the data of the experiments. Q-YT and ML wrote the manuscript. All authors contributed to manuscript revision.

## Conflict of Interest

The authors declare that the research was conducted in the absence of any commercial or financial relationships that could be construed as a potential conflict of interest.
