# Supplementary figures and images for: Adiponectin Mediates the Protection of H2S Against Chronic Restraint Stress-Induced Cognitive Impairment via Attenuating Hippocampal Damage
Source: Front Behav Neurosci. 2021 May 5;15:623644. doi: 10.3389/fnbeh.2021.623644 (PMC8131522; doi:10.3389/fnbeh.2021.623644)

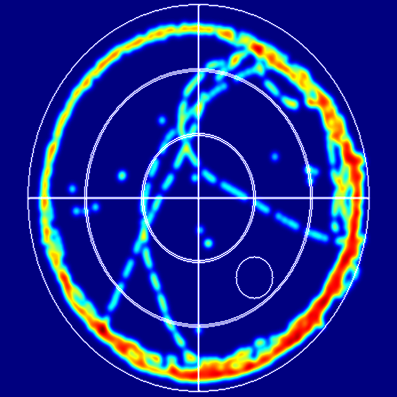

Supplement: Supplementary file 1 [file Data_Sheet_1.ZIP › Morris water maze test/The training phase and acquisition phase/Route of rats in 1 st in training phase/Anti-Acrp30 group.bmp]

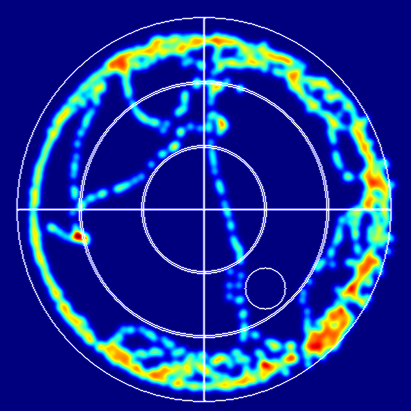

Supplement: Supplementary file 1 [file Data_Sheet_1.ZIP › Morris water maze test/The training phase and acquisition phase/Route of rats in 1 st in training phase/Control group.bmp]

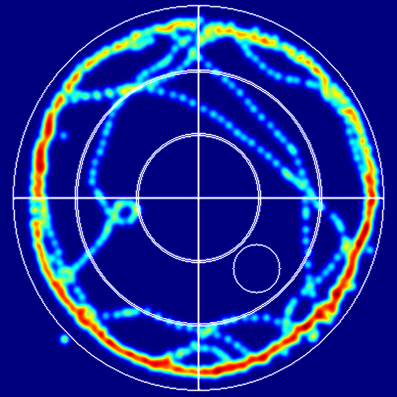

Supplement: Supplementary file 1 [file Data_Sheet_1.ZIP › Morris water maze test/The training phase and acquisition phase/Route of rats in 1 st in training phase/CRS group.bmp]

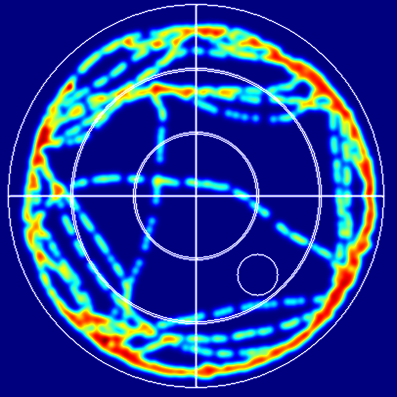

Supplement: Supplementary file 1 [file Data_Sheet_1.ZIP › Morris water maze test/The training phase and acquisition phase/Route of rats in 1 st in training phase/CRS+NaHS+Anti-Acrp30 group.bmp]

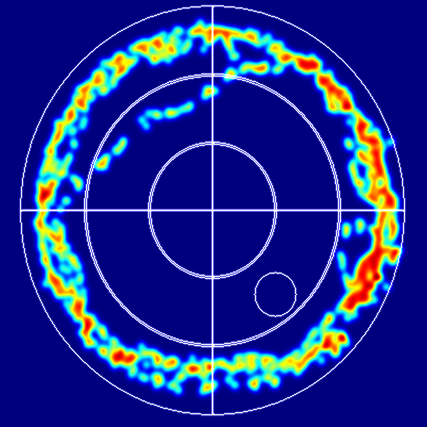

Supplement: Supplementary file 1 [file Data_Sheet_1.ZIP › Morris water maze test/The training phase and acquisition phase/Route of rats in 1 st in training phase/NaHS+CRS group.bmp]

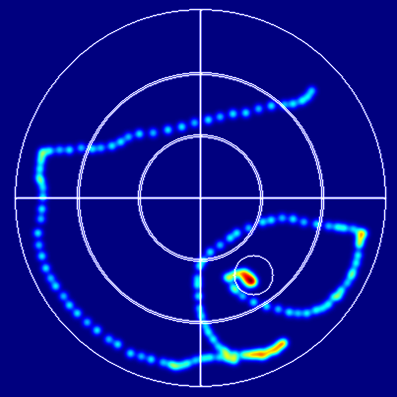

Supplement: Supplementary file 1 [file Data_Sheet_1.ZIP › Morris water maze test/The training phase and acquisition phase/Route of rats in 5 th in training phase/Anti-Acrp30 group.bmp]

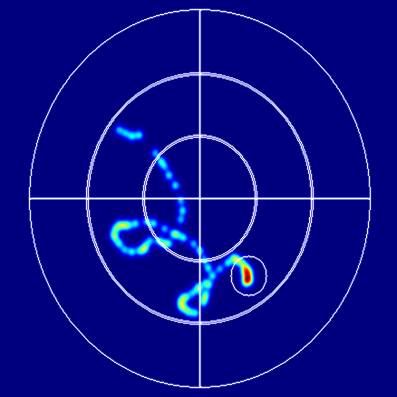

Supplement: Supplementary file 1 [file Data_Sheet_1.ZIP › Morris water maze test/The training phase and acquisition phase/Route of rats in 5 th in training phase/Control group.bmp]

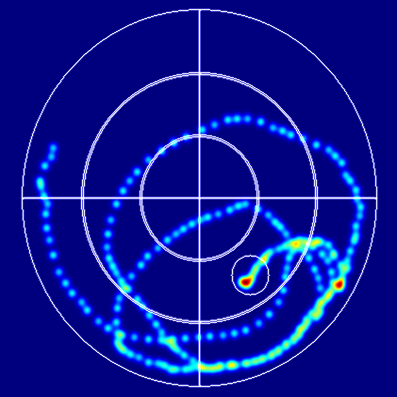

Supplement: Supplementary file 1 [file Data_Sheet_1.ZIP › Morris water maze test/The training phase and acquisition phase/Route of rats in 5 th in training phase/CRS group.bmp]

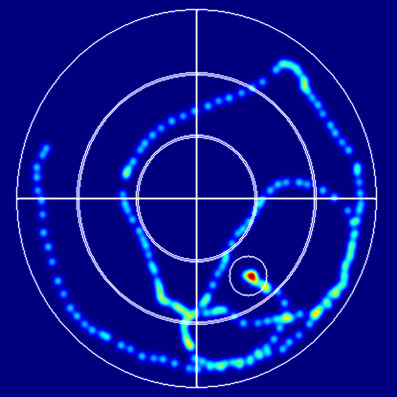

Supplement: Supplementary file 1 [file Data_Sheet_1.ZIP › Morris water maze test/The training phase and acquisition phase/Route of rats in 5 th in training phase/CRS+NaHS+Anti-Acrp30 group.bmp]

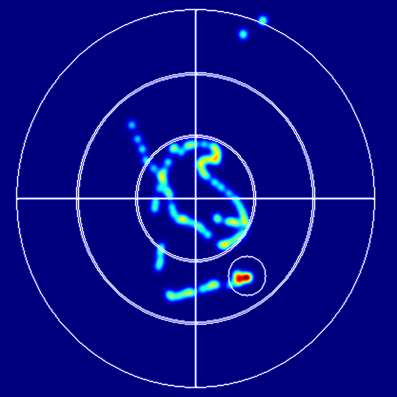

Supplement: Supplementary file 1 [file Data_Sheet_1.ZIP › Morris water maze test/The training phase and acquisition phase/Route of rats in 5 th in training phase/NaHS+CRS group.bmp]
